# Prion Pathogenesis Revealed in a Series of the Special Issues “Prions and Prion Diseases”

**DOI:** 10.3390/ijms23126490

**Published:** 2022-06-10

**Authors:** Suehiro Sakaguchi

**Affiliations:** Division of Molecular Neurobiology, The Institute for Enzyme Research (KOSOKEN), Tokushima University, 3-18-15 Kuramoto, Tokushima 770-8503, Japan; sakaguchi@tokushima-u.ac.jp

Prion diseases are a group of devastating neurodegenerative disorders, which include Creutzfeldt–Jakob disease (CJD) in humans, and scrapie and bovine spongiform encephalopathy (BSE) in animals [1,2]. No therapeutic interventions have been developed for these diseases. A series of the Special Issues “Prions and Prion Diseases” and “Prions and Prion Diseases 2.0” aimed to help increase our understanding of the pathogenic mechanism of prion diseases, and thereby contribute to the development of therapeutic interventions against prion diseases. Thanks to many contributions, this Special Issue has successfully provided valuable insights into various pathogenic aspects of prion disease.

Prion diseases are caused by the conformational conversion of the cellular isoform of prion protein, designated PrP^C^, into the relatively protease-resistant amyloidogenic isoform, PrP^Sc^, which consequently aggregates and forms proteinaceous infectious particles or prions [1,2]. It remains controversial whether the conversion of PrP^C^ into PrP^Sc^ results in the loss-of-function of PrP^C^, therefore evoking neuronal dysfunctions eventually causing prion diseases. Mice devoid of PrP^C^ (*Prnp^0/0^*) have been shown not to autonomously develop prion disease [3]. However, abnormal neuropathological phenotypes similar to those often observed in patients with these diseases, such as impaired learning behaviors, altered circadian rhythm and sleep, demyelination in the spinal cords and peripheral nerves, and abnormal olfactory functions, have been reported in *Prnp^0/0^* mice [4,5,6,7,8]. These results suggest the possibility that these pathological conditions observed in prion diseases might be attributable to the loss-of-function of PrP^C^. PrP^C^ is a glycosylphosphatidylinositol-anchored membrane glycoprotein expressed most abundantly in the brains, particularly by neurons, and, to a lesser extent, in other non-neuronal tissues [1,2]. In this Special Issue, Martellucci et al. reviewed the signaling pathway of PrP^C^ in the self-renewal and proliferation of tissue-resident stem cells and the neuronal differentiation of neuronal stem cells [9]. Prado et al. presented the cell signaling of PrP^C^ in cell motility [10]. Mahabadi and Taghibiglou comprehensively profiled a large number of reported PrP^C^-interacting molecules and discussed the various cellular functions of PrP^C^ [11]. However, the normal function of PrP^C^ still remains elusive. Further studies are needed to identify the normal function of PrP^C^ and answer the question of whether the functional deficiency of PrP^C^ could be involved in the pathogenesis of prion diseases.

The neuron-specific conditional knockout of PrP^C^ in mice has been reported not to develop prion disease, even after intracerebral inoculation with prions, although PrP^Sc^ was abundantly accumulated in glial cells and marked gliosis was observed in their brains [12], suggesting that expression of PrP^C^ in neurons is essential for prions, or PrP^Sc^, to exert neurotoxicity causing prion diseases. We have shown that the post-Golgi vesicular trafficking of certain membrane proteins was disturbed in prion-infected neuronal cells and mouse brains [13]. Other investigators also reported that genetic prion disease-associated PrP mutants impaired the post-Golgi vesicular trafficking in cultured cells and primary neurons of transgenic mice [14,15]. Furthermore, endo-lysosomal trafficking important for lysosomal maturation was reported to be defective in prion-infected cells; therefore, lysosomal maturation and lysosomal protein degradation are impaired in the cells [16]. It is thus conceivable that PrP^Sc^ or mutant PrPs in neurons might target the intracellular vesicle trafficking system, eventually causing neuronal dysfunctions associated with prion diseases. In this series, Cherry and Gilch reviewed the possible mechanism of the PrP^Sc^-associated vesicular trafficking defect by highlighting how the Rab GTPase family-associated vesicular trafficking system is impaired in prion-infected neurons [17]. Alves et al. also discussed the mechanism of the inter- and intra-cellular trafficking of PrP^C^ and PrP^Sc^ [18].

The PrP conversion mechanism remains to be fully determined. During conversion into PrP^Sc^, PrP^C^ undergoes marked conformational changes in the 2/3 C-terminal region, forming a protease-resistant structure [1,2]. It has been suggested that the structural transition of α-helices to β-sheets in the C-terminal region is a key mechanism underlying the conversion of PrP^C^ into PrP^Sc^ [1,2]. Cryo-electron microscopic analysis revealed a parallel in-register intermolecular β-sheet structure consisting of a large number of β-sheets as the PK-resistant structure of PrP^Sc^ [19,20]. Many structure-and-function studies for PrP^C^ to convert into PrP^Sc^ have been performed by reconstituting *Prnp^0/0^* mice with transgenes encoding PrP molecules with deletions or point mutations. In this series, *Prnp^0/0^* mice expressing PrP lacking residues 91–106 were shown to be resistant to RML, 22L, and FK-1 prions, while they were marginally susceptible to BSE prions, suggesting that residues 91–106 could be involved in the prion strain-dependent conversion of PrP^C^ into PrP^Sc^ [21]. Hara and Sakaguchi also comprehensively reviewed the reported structure-and-function studies for PrP^C^ by focusing on the role of the N-terminal region of PrP^C^ in the conversion into PrP^Sc^ [22]. Carlson and Prusiner discussed how different protein conformations of PrP^Sc^ can form different prion strains with strain-specific pathogenic properties [23].

In human prion diseases, sporadic CJD with unknown etiologies is the most prevalent form, accounting for 85–90% of cases [24]. Moreover, 10–15% of cases belong to genetic prion diseases, which causatively link to specific mutations in the PrP gene, or *Prnp* [25]. The remaining small number of cases, including those of BES-transmitted variant CJD, iatrogenic CJD, and kuru, are acquired prion diseases caused by prion infection events [26]. In this series, we introduced a report which demonstrated that infection with a neurotropic strain of influenza A virus induced the de novo conversion of PrP^C^ into PrP^Sc^ and produced infectious prions in mouse neuroblastoma N2a cells, shedding light on the possibility that infection with influenza A viruses or other related viruses in neurons might be a cause of or associate with the pathogenesis of sporadic CJD [27,28]. We also reviewed transgenic mouse models reported for genetically associated prion diseases [28].

This Special Issue included other reports, such as those describing the identification of ethanolamine as a novel anti-prion agent or the usefulness of the real-time quaking-induced conversion assay in diagnosis of sCJD [29,30]. To further clarify the pathogenic mechanism of prion diseases and to develop clinically effective treatments against the diseases, the Special Issue “Prions and Prion Diseases 3.0” has been set up. Many contributions, which facilitate further understanding of the pathogenic mechanism of prion diseases and promote the developments of therapeutic interventions against the diseases, are welcomed.
